# A randomized prospective controlled trial comparing the laryngeal tube suction disposable and the supreme laryngeal mask airway: the influence of head and neck position on oropharyngeal seal pressure

**DOI:** 10.1186/s12871-016-0237-7

**Published:** 2016-10-06

**Authors:** Mostafa Somri, Sonia Vaida, Gustavo Garcia Fornari, Gabriela Renee Mendoza, Pedro Charco-Mora, Naser Hawash, Ibrahim Matter, Forat Swaid, Luis Gaitini

**Affiliations:** 1 Anesthesiology Department, Bnai Zion Medical Center and Bruce and Ruth Rappaport Faculty of Medicine, Technion, Israel Institute of Technology, Haifa, Israel; 2 Surgery Department, Bnai Zion Medical Center and Bruce and Ruth Rappaport Faculty of Medicine, Technion, Israel Institute of Technology, Haifa, Israel; 3 Anesthesiology Department, Penn State College of Medicine, Hershey, PA USA; 4 Anesthesiology Department, Hospital Universitario Italiano, Buenos Aires, Argentina; 5 Anesthesiology Department, Hospital Universitario de Valencia, Valencia, Spain; 6 International Program of Teaching and Investigation in Airway Management – FIDIVA, Haifa, Israel

**Keywords:** Laryngeal tube, Supreme laryngeal mask airway, Oropharyngeal seal pressure

## Abstract

**Background:**

The Laryngeal Tube Suction Disposable (LTS-D) and the Supreme Laryngeal Mask Airway (SLMA) are second generation supraglottic airway devices (SADs) with an added channel to allow gastric drainage. We studied the efficacy of these devices when using pressure controlled mechanical ventilation during general anesthesia for short and medium duration surgical procedures and compared the oropharyngeal seal pressure in different head and-neck positions.

**Methods:**

Eighty patients in each group had either LTS-D or SLMA for airway management. The patients were recruited in two different institutions. Primary outcome variables were the oropharyngeal seal pressures in neutral, flexion, extension, right and left head-neck position. Secondary outcome variables were time to achieve an effective airway, ease of insertion, number of attempts, maneuvers necessary during insertion, ventilatory parameters, success of gastric tube insertion and incidence of complications.

**Results:**

The oropharyngeal seal pressure achieved with the LTS-D was higher than the SLMA in, (extension (*p*=0.0150) and right position (*p*=0.0268﻿﻿ at 60 cm H_2_O intracuff pressures and nearly significant in neutral position (*p* = 0.0571). The oropharyngeal seal pressure was significantly higher with the LTS-D during neck extension as compared to SLMA (*p*= 0.015). Similar oropharyngeal seal pressures were detected in all other positions with each device. The secondary outcomes were comparable between both groups. Patients ventilated with LTS-D had higher incidence of sore throat (*p* = 0.527). No major complications occurred.

**Conclusions:**

Better oropharyngeal seal pressure was achieved with the LTS-D in head-neck right and extension positions , although it did not appear to have significance in alteration of management using pressure control mechanical ventilation in neutral position. The fiberoptic view was better with the SLMA. The post-operative sore throat incidence was higher in the LTS-D.

**Trial registration:**

ClinicalTrials.gov ID: NCT02856672, Unique Protocol ID:BnaiZionMC-16-LG-001, Registered: August 2016.

## Background

**Fig.1:**
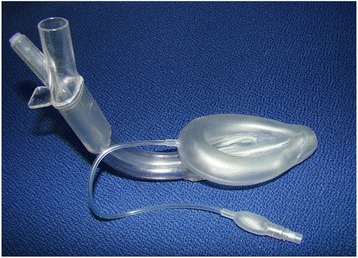
Supreme Laryngeal Mask Airway

**Fig. 2:**
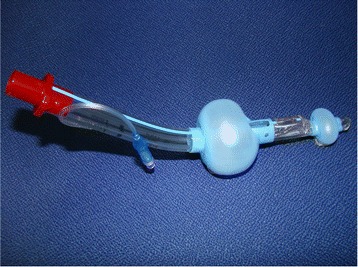
Laryngeal Tube Suction Disposable

Laryngeal Tube Suction Disposable, LTS-D (VBM Medizintechnik GmbH, Sulz, Germany) [1, 2] and Supreme Laryngeal Mask Airway, SLMA (Intavent Orthofix, Maidenhead, UK) [3, 4] are second generation, single-use, supraglottic airway devices (SADs), with added gastric access, for use in spontaneously and mechanically ventilated patients undergoing general anesthesia (Fig. 1) Supreme Laryngeal Mask Airway and (Fig. 2) Laryngeal Tube Suction Disposable.

The effectiveness of the LTS-D and the SLMA has been well established [5–11]; however, the oropharyngeal seal pressure of both devices in different head/neck positions and the performance of these devices using positive pressure ventilation have not been evaluated. Changing the head/neck position can alter the sealing capabilities of the SAD.

To our best knowledge there are no studies comparing the oropharyngeal seal pressure in different head and neck position when using these devices.

Our study compared the LTS-D with the SLMA, hypothesizing that the two devices would provide adequate oropharyngeal seal pressure in different head and neck positions and perform similarly during pressure controlled ventilation in neutral position, despite differences in their structural design.

## Methods

One hundred sixty patients, ASA physical status I and II weighing between 50 and 100 kg, with normal airways, undergoing general anesthesia for minor elective surgical procedures in supine position were randomly assigned to have either a LTS-D or a SLMA for airway management.

Two hospitals in two different countries (Bnai Zion Medical Center, Haifa, Israel and Italiano Hospital in Buenos Aires, Argentina) participated in the study. IRB approval was obtained separately for each institution and written informed consent was obtained from each of the study subjects. The patients were blinded to their device type assignment. Each study center conducted eighty cases with even randomization for the type of airway device. Randomization was performed using a computer generated list and by opening a sealed envelope immediately before induction. The surgery types were from a wide range of elective minor general surgery, orthopedic, urologic, gynecologic, and plastic surgery of short and moderate duration.

Exclusion criteria were: age <18 years, weight <50 kg, or >100 kg, body mass index >35 kg/m^2^, cervical spine disease limiting neck movement, a known difficult airway, intrinsic lung disease and patients having active gastro esophageal reflux disease. The following demographic data were collected for each patient: sex, age, height, weight, body mass index, type of operation, and total time of anesthesia. Two attending anesthesiologists in each medical center participated in the trial. Each had previously performed more than 50 insertions of the LTS-D and SLMA. Midazolam 0.05 mg/kg IV and IV fentanyl 2 mcg/kg were administered during a 3 min period of time during preoxygenation. General anesthesia was induced with IV propofol 2 mg/kg and neuromuscular blockade was achieved with IV rocuronium bromide 0.6 mg/kg. After induction, sevoflurane end-tidal concentration up to 1.5 % in 33 % oxygen and 66 % nitrous oxide was started and ventilation was controlled by facemask was controlled by facemask for three min, and then the SAD was inserted.

A size 4 or 5 LTS –D and size 4 or 5 SLMA was used according to manufacturer’s recommendations. In accordance with these recommendations the cuffs were inflated to 60 cm H_2_O immediately after insertion, using a cuff pressure gauge (VBM Medizintechnik GmbH, Sulz, Germany).

The airway was judged to be effective and adequate if an expiratory volume of at least 6 mL/kg was obtained during gentle manual ventilation, at a peak airway pressure of at least 15 cm of H_2_O, no oral leak as judged by stethoscope auscultation over the neck, and a normal square-wave capnograph trace. The expiratory volume data and the square wave capnograph trace were obtained using the integrate spirometer and capnograph of the S/5™ Anesthesia Delivery Unit (EDU) (Datex-Ohmeda, Helsinki, Finland).

The total time to achieve an effective airway was measured as the time after the anesthesiologist removed the facemask until the square-wave capnograph tracing was observed after insertion. Two attempts at insertion were permitted. If unsuccessful (as judged by an inability to insert the device or by a total lack of ventilation), we planned to secure the airway using an endotracheal tube.

If after the insertion of the device the airway was judged to be inadequate by the above criteria, but some ventilation was obtained, further maneuvers were allowed to properly position the device. These adjustments included minor interventions: (adjusting head or neck position, applying jaw lift or changing depth of insertion) or major interventions (reinsertion of the device, or changing the size).

Insertion of the devices was classified as 1 = easy, 2 = moderate, 3 = difficult and 4 = impossible, as evaluated by the anesthesiologist.

Once an effective airway was achieved, oropharyngeal cuff seal pressures were obtained by closing the expiratory valve of the anesthesia circuit with a fixed gas flow rate of 3 L/min and noting the airway pressure at which equilibrium was reached. The maximum allowed airway pressure during this evaluation period was 40 cm H_2_O [12]. The oropharyngeal cuff seal pressure was obtained in neutral position (occiput on standard firm pillow 7 cm in height), maximal flexion (chin touching the thorax) maximal extension and maximal lateral left and right head/neck rotation. Before checking the oropharyngeal seal pressure in each different head and neck position the intra cuff pressure was re-checked and adjusted to 60 cm H_2_O if necessary, in order to standardize the measurement conditions. This way, the oropharyngeal seal pressure was only influenced by the change of the anatomical position.

During the maintenance of the anesthesia the patients’ lungs were initially ventilated with 17 cm H_2_O pressure control ventilation, a respiratory rate of 12/min, and adjustments made to maintain the ETCO_2_ to 35–40 mmHg. Respiratory variables were measured using a Datex AS-5 monitor.

The fiberscope score was determined in all cases in neutral position by passing a pediatric flexible bronchoscope (Storz, Germany, 3.1 mm) through the airway tube of the devices to a level of the one of two ventilation apertures of the LTS-D or to the unique ventilation aperture of the SLMA. In this position, the operator could redirect the tip of the fiberoptic bronchoscope in order to obtain best possible glottic view.

We used a previously described scoring system scoring the fiberoptic view [13]. The SLMA has the drain tube on the central part of the airway channel. Therefore, the fiberoptic scope can pass on either side of the drain tube until reaching the ventilation aperture situated between the epiglottis fins. The LTS-D has a separate drain channel, so that the fiberoptic scope is passed in the middle of the airway channel and the view could be obtained through the two frontal apertures of the device. The fiberoptic score was ranged from grade 4 (full view of arytenoids and glottis), 3 (arytenoids and glottis partly visible), 2(view of arytenoids, glottis or epiglottis), and 1 (no part of larynx identifiable).

A single attempt was made to pass a lubricated (K-Y gel, Johnson and Johnson, USA) 18 gauge - French gastric tube through the LTS-D and a 16-French gauge gastric tube through the drain tube of the SLMA. Placement of the gastric tube in the stomach was confirmed by aspiration of gastric contents or synchronous injection of air and epigastric auscultation. After successful insertion, the gastric tube was removed.

The following data were recorded every 5 min commencing after checking the oropharyngeal seal pressure until the administration of the reversal of the muscular relaxant: oxygen saturation, inspired and expired tidal volumes, respiratory rate, fraction of inspired oxygen, ETCO_2_, peak inspiratory pressure, plateau pressure, and PEEP.

The integrity of the flow volume loops to monitor for leak was controlled during all the procedure. At the end of the surgery sevoflurane and nitrous oxide were discontinued and the patients were allowed to breathe 100 % O_2_. Reversal of the neuromuscular blockade was obtained with neostigmine and atropine.

The airway device was removed when the patients could open their mouths to command and after a train-of four count of four was obtained.

Each device was examined for the presence of blood and the mouth was inspected for dental or mucosal trauma. Patients were interviewed to determine the incidence and severity of postoperative airway related adverse events. Non-leading questions were asked by a blinded research assistant after release of the patient from the Post Anesthetic Care Unit (PACU) and 24 h postoperatively.

Perioperative adverse events were graded: mild = coughing or gagging on insertion, hiccups, gastric insufflations; moderate = bronchospasm, airway obstruction, blood staining of the device, oral or tongue pain, sore throat, hoarseness, difficulty in swallowing, sore neck, mandibular pain, dysphasia and dysphonia; severe = hypoxia, regurgitation, aspiration, dental trauma, soft tissue trauma, gross blood-staining of the device.

### Statistical analysis

For the demographic continuous variables (age, weight, height and BMI) means and standard deviations were calculated. The results of the demographic continuous variable between the two study groups (Supreme vs LTS) were analyzed by the 2 sample T-test for differences of mean.

For the categorical variables (gender, ASA, device insertion categorical parameters and postoperative complications), numbers and percentages were calculated. The distributions for the categorical variables between the two study groups were compared and analyzed by the Chi square test (a parametric test) or by Fisher-Irwin exact test (a non-parametric test for small numbers).

For the continuous of seal pressure variables means, standard deviations and ranges were calculated. The results of the leak pressure continuous variable between the two study groups (SLMA vs. LTS-D) were analyzed by the two sample T-test for differences of mean.

The results of the leak pressure continuous variable comparing neutral to each of the other head/neck positions in each of the two study groups were analyzed by the T-test paired.

For the respiratory continuous variables of inspirium, expirium, saturation, ETCO_2_, peak inspiratory pressure, PEEP the average of the 12 repeated measurements (every 5 min in first the hour) for each patient was calculated and then means and standard deviations were computed. The results of the respiratory continuous variables between the two study groups (Supreme vs. LTS) were analyzed by the two sample T-test for differences of mean.

All statistical tests were analyzed to a significance level of 0.05.

Based on previous studies [14, 15], the sample size was calculated to find a difference of 5 cm of H_2_O in the oropharyngeal seal pressure between the two devices with standard deviation of 8 cm H_2_O. for a type I error of 0.05 and power of 0.88. This calculation allowed us to establish sample size of 40 patients in each group being (80 per each medical center with a total of 160 patients).

## Results

There was no difference in the demographic and surgical data between the groups, Table 1. LTS-D and the SLMA were inserted successfully in all patients requiring one attempt in 87.5 and 86.2 % of patients for SLMA and LTS-D respectively and a second attempt in 12.5 and 13.8 % of patients for SLMA and LTS-D respectively (*p* = 0.8150). The insertion time was 31.6 ± 12 and 29.4 ± 12.8 seconds for the SLMA and LTD-S respectively (*p* = 0.2680).

**Table 1:** Demographic and surgical data

	SLMA (*n* = 80)	LTS-D (*﻿n﻿*= 80)	*p* value
Age (years)	55.1 ± 11.3	55.5 ± 13.3	^*†*^0.8080
Weight (kg)	68.5 ± 8.3	69.3 ± 10.7	^*†*^0.6136
Height (cm)	167 ± 9	167 ± 8	^*†*^0.9548
BMI (kg/m^2^)	24.7 ± 2.4	25.0 ± 3.4	^*†*^0.5067
Gender (M/F)	29/51 36/64 %	32/48 40/60 %	^*├*^0.6250
ASA(I/II)			
I	17 (21)	14 (18)	^*├*^0.5480
II	63 (79)	66 (82)	
Duration of anesthesia (min)	45.2 ± 20.9	48.3 ± 22.7	^*†*^0.3725

Data are mean ± SD or numbers (%)

*P* value by ^*†*^2- sample T-test for differences of mean or ^*├*^chi square test; *p* >0.05 (NS)

Significantly less minor airway interventions were needed with the SLMA (*p* = 0.005). Data describing the ease of insertion and airway interventions is described in Table 2.

**Table 2:** Insertion data

	SLMA (*n* = 80)	LTS-D (*n* ﻿= 80)	*p* value
Ease level			
1	64 (80.0)	58 (72.5)	^*┴*^0.5900
2	11 (13.8)	15 (18.8)	
3	5 (6.2)	7 (8.7)	
Minor intervention			
No intervention	35 (43.8)	19 (23.8)	^*├*^0.0050**
Adjusting head/neck position	16 (20.0)	33 (41.2)	
Jaw lift or changing depth of insertion	29 (36.2)	28 (35.0)	

Data are mean ± SD or numbers (%)

*P* value by ^*├*^chi square test; or ^*┴*^Fisher exact test; **p ≤0.01 (Sig) or *p* >0.05 (NS)

The oropharyngeal seal pressure of the LTS-D was higher that the SLMA in ﻿extension (*p*=0.0150) and right position﻿ (*p*=0.0268﻿ at 60 cm H2O intracuff pressure﻿. This probably reflects the different mechanism of seal of the two devices. The cuff of the SLMA surrounds the laryngeal inlet in longitudinal position and forms a seal with the periglottic tissues, whereas the LTS-D forms a seal by exerting pressure against all the circumference of the pharyngeal mucosa.

No significant differences have been detected between devices in all other positions, Table 3.

**Table 3:** Oropharyngeal seal pressure

Leak pressure	SLMA (*n* = 80)	LTS-D (*n*﻿= 80)	*p* value
Neutral (cmH_2_O)	33 ± 6 (19–40)	35 ± 6 (22–40)	^*†*^0.0571
Extension (cmH_2_O)	31 ± 5 (17–40)	33 ± 5 (20–40)	^*†*^0.0150*
Flexion (cmH_2_O)	35 ± 6 (19–40)	35 ± 5 (21–40)	^*†*^0.4711
Right (cmH_2_O)	32 ± 5 (19–40)	34 ± 5 (19–40)	^*†*^0.0268*
Left (cmH_2_O)	32 ± 5 (18–40)	34 ± 5 (18–40)	^*†*^0.1140
Balloon pressure (cmH_2_O)	60 ± 0.2 (58–60)	60.0 ± 0 (60–60)	^*†*^0.3188

Data are mean ± SD (range)

*P* value by ^*†*^2- sample T-test for differences of mean *p ≤0.05 (Sig) or *p* >0.05 (NS)

When compared with neutral position the oropharyngeal seal with SLMA was significantly higher with head/neck flexion (*p* = 0.0007) and significantly lower with head/neck extension (0.0002) and right rotation. (0.0480). When compared with neutral position the oropharyngeal seal pressure for the LTS-D was significantly lower with head/neck extension. (0.0038).

The mean peak inspiratory pressure and plateau pressure in patients with SLMA was significantly lower than in patients with LTS-D (*p* = 0.006) and (*p* = 0.008).

Compared with the neutral position, the leak pressure for the SLMA was significantly higher from extension (0.0002) and from right position (0.0480), and significantly lower from flexion (0.0007).

Compared with the neutral position, the leak pressure for the LTS-D was significantly higher from extension (0.0038).

No significant differences have been detected between devices for all other respiratory parameters (Table 4).

**Table 4:** Respiratory data

	SLMA (*n* = 80)	LTS-D (*n* = 80)	*p* value
Ventilation inspirium (ml)	501 ± 125	524 ± 138	^*†*^0.2787
Ventilation expirium (ml)	444 ± 110	475 ± 123	^*†*^0.0965
Ventilation delta (ml)	57 ± 35	48 ± 26	^*†*^0.0792
O_2_ Saturation (%)	99 ± 0.8	99 ± 0.8	^*†*^0.8632
ETCO_2_ (mmHg)	33 ± 3	34 ± 4	^*†*^0.4356
Peak (cmH_2_O)	16 ± 3	18 ± 4	^*†*^0.0060**
Peep (cmH_2_O)	2 ± 0.7	2 ± 0.6	^*†*^0.2631
P plat (cmH_2_O)	11 ± 2	12 ± 2	^*†*^0.0080**

Data are mean ± SD

*P* value by ^*†*^2- sample T-test for differences of mean; **p ≤0.01; *p* >0.05 (NS)

There was a significant difference in fiberoptic view score favoring the SLMA. Fiberoptic position (4/3/2/1/) was 25/34/15/6 for the SLMA and 15/25/11/29 for the LTS-D (*p* = 0.0001).

Upper airway trauma, as evaluated by the presence of blood staining of the devices after their removal was not significant statistically between the two devices.

Gastric tube insertion was successful in all patients in both groups.

In PACU, there was no significant difference between the groups regarding the post-operative complications. (Sore throat: 12 % in the SLMA group (95 % C.I: ±7.8) and 14 % in the LTS-D group, (95 % C.I.: ±8.3) (*p* = 0.668), dysphagia: 0 % in the SLMA group and in the LTS-D 2 % group (*p* = 0.497), (95 % C.I.: ±3.4) dysphonia: 0 % in the SLMA group and 3 % in the LTS-D group (95 % C.I.: ±4.2) (*p* = ^˫^0.245).

A significantly higher incidence of post-operative sore throat was detected in patients with LTS-D (26.3 %) (95 % C.I.: ±9.7) as compared to patients with SLMA (6.3 %) (95 % C.I.: ±5.3) after 24 h (*p* = 0.001).

The percentage of patients with at least one complication was 13 % in the SLMA group and 18 % in the LTS-D group (*p* = 0.317).

Gastric tube insertion was successful in all patients in both groups.

## Discussion

The main finding of this study was that the oropharyngeal seal pressure for the LTS-D is higher that the SLMA in right an﻿d head- neck extended position, at 60 cm H_2_O intracuff pressures.

Even though there was statistically significant difference favoring the LTS – D, this finding has no clinical significance as both devices proved to have an excellent seal pressure.

Our results are consistent with previous studies reporting high seal pressure with both devices [6, 10, 16–18]. Genzwuever et al [10] found a oropharyngeal seal pressure of 33.1 cm H_2_O using LTS II, a device similar to LTS –D.

However, there are also reports of a very low oropharyngeal seal pressure for both devices [15, 19]. Kikuchi et al. [15] found an oropharyngeal seal pressure as low as 16 cm H_2_O with the LTS II, which the author attributes to the Asian ethnicity of the participants. Tham et al. [19], in a crossover study also reports a low oropharyngeal seal pressure for the SLMA (19.6 cm H_2_O).

The value of the fiberoptic anatomical position assessment of a SAD in predicting a functional oropharyngeal seal is controversial [20, 21, 22]. The better fiberoptic score we obtained with the SLMA may have a potential benefit when considering an endotracheal tube placement thought the device. The vocal cords could not be visualized in 36.3 % of the patients with LTS-D, however in all these patients ventilation was adequate, confirming findings in a previous report that there is no correlation between the adequacy of ventilation and a low fiberoptic score [6].

We found an increased oropharyngeal seal pressure in the SLMA and in the LTS-D during neck flexion. Our results are in accordance with the Brimacombe et al. [23] reports on the influence of the head and neck flexion and extension on the oropharyngeal seal pressure. A reduction or increase of approximately 25 % in pharyngeal volume during head and neck extension or flexion may increase or decrease the oropharyngeal seal pressure [23]. Early reports with the Classic LMA described an increased oropharyngeal seal pressure during neck flexion and decreased during extension [24, 25]. Neck flexion causes a reduction in the antero posterior pharyngeal diameter and with the better seal provided by the SLMA the oropharyngeal seal pressure is increased. Neck extension increases the pharyngeal antero-posterior diameter by raising the laryngeal inlet, leading to a reduced contact of the SLMA cuff with the mucosa and therefore a drop in the oropharyngeal seal pressure. The oropharyngeal seal pressure decreased for both devices in right and left head and neck rotation suggesting that the contact with the pharyngeal mucosa could be affected by the change in the antero-posterior and lateral diameter. Similar to Park et al. [26], our study showed that the LTS-D can maintain an acceptable oropharyngeal seal pressure after extension, flexion and rotation of the head and neck. Even though we found statistically significant decreases of the oropharyngeal seal pressure in right head/neck and extension position, the seal pressures remained high enough to potentially allow effective mechanical ventilation. We cannot specifically comment of the ventilation adequacy in this positions as we did not address this question in our study. Surgeries in different head/neck positions using SAD’s to secure the airway are frequently performed and additional studies need to be performed to check the effectiveness of the devices in these positions.

In our study, we found both the LTS-D and the SLMA easy and quick to insert, with an equally high success on the first attempt. However the data concerning the insertion of both devices is different in comparison with other studies.

The insertion time for the LTS-D in our study was longer than that reported by Russo et al. [16] with the LTS-D, (14 s) and from Mihai et al. [6] with the LTS II, (15 s), and faster than the one of Schalk et al. [27] performed by paramedics and emergency physicians using LTS-D, (45 s) and Kikuchi T et al. [15] using the LTS II, (40 s). The insertion time for the SLMA was longer in comparison with other studies. Verghese et al. [4] reported an insertion time of 15 s and Cook et al [28] 18 s, however it was faster than the insertion time for SLMA was described by Zhang et al. 38 s [29]. We believe that the differences in the insertion time could be related to the investigators experience and different definition to define an effective airway.

Despite the high percentage of the maneuvers necessary to optimize the position of the device, the success rate after two insertion attempts was 100 % reflecting the excellent clinical effectives of both devices. Cook et al. [28] reported 30 maneuvers necessary to optimize the airway patency in 24 patients.

Most studies report no failures during inserting the LTS-D [6, 10] and SLMA [18, 28, 30]; however, success rate of as low as 70 % with the LTS-D has also been reported [16]. Both devices provided optimal oxygenation and ventilation during pressure control ventilation.

We found a higher peak pressure inspiratory pressure with the LTS-D. Previous studies with the first version of the Laryngeal Tube Suction (LTS) (with only two ventilation orifices) reported a high inspiratory pressure attributed to the narrow size of the ventilations holes or to the obstruction by the soft tissue causing increased resistance to gas flow [11, 31]. The LTS-D used in the present study has four additional ventilation orifices to improve ventilation. Our results confirming those of Russo et al. [16] proving that the resistance to gas flow is still high and probably additional improvement of the ventilation outlet is still needed.

We had a high success rate in passing a gastric tube trough the gastric port of both devices. The LTS-D allows passage of the gastric drain tube number 18F whereas the largest size tube that can be used for a SLMA is 16F. We did not find a correlation between the successful insertion of a gastric tube and the fiberoptic score, even though that was previsously reported for the Proseal Laryngeal Mask Airway and has been associated with a good fiberoptic score [32, 33]. The reported sore throat related to LTS-D use varies largely from 7 to 71 % [6, 10, 16]. Probably depending on the level of the providers experience with the device. We found 14 % of patients in the LTS-D group complaining of sore throat when leaving PACU. Our incidence of sore throat with the SLMA is higher than the results reported by others studies [18, 31, 33] (8–15 %) but similar with the that reported by Bermann et al. [34].

However, the causes of postoperative adverse events such as sore throat after general anesthesia using supraglottic devices are multifactorial, with the overall incidence influenced by the depth of anesthesia at the time of insertion, the method of insertion [35], the number of insertion attempts [36] the duration of the anesthesia [36], the mode of ventilation used [37], and the type of postoperative analgesia provided [38]. Similar to Cook et al [28] and Mihai et al [6] we found symptoms of upper airway trauma after 24 h, 6. 3 % in the SLMA group and 26.3 % of patients in the LTS group complained for sore throat. The incidence of sore throat after 24 h was higher than it the PACU, presumably because of the masking effect of analgesics administered in the immediate post-operative period. Twenty four hours after the discharge of PACU, 8.8 % of SLMA patients group and 31.3 % of LTS-D patients group had at least one minor perioperative complication event suggesting that the SLMA is less traumatic that the LTS-D.

Our study has several limitations. Although the investigators were experienced with both devices, the large prior experience with all types of the LMAs may have given the SLMA a possible advantage. In addition, the operators were obviously unblinded to the type of the SAD so that bias could not be excluded. The devices were inserted three minutes after the administration of the neuromuscular blocking agent however, we did not use neuromuscular blockade monitoring to confirm to ensure similar SAD placement conditions.

Our results may not be applicable to patients with spontaneous ventilations.

We didn’t ventilate the patients in the different head and neck positions, so we cannot know the performance of the devices during mechanical ventilation despite the good seal pressure measured. Cuffs constructed from PVC are less susceptible to nitrous oxide diffusion [39]. However, as we did not check the intracuff pressures throughout the procedure, we cannot exclude possible nitrous oxide diffusion that could affect the seal pressure of the devices.

## Conclusions

Better oropharyngeal seal pressure was achieved with the LTS-D in head-neck right and extension positions, although it did not appear to have significance in alteration of management using pressure control mechanical ventilation in neutral position.

## Abbreviations

ASA: American anesthesiology society
BMI: Body mass index
EDU: Anesthesia delivery unit
ETCO_2_: End tidal carbon dioxide
LMAU: Laryngeal mask airway unique
LTS II: Laryngeal tube suction II
LTS-D: Laryngeal tube suction disposable
PACU: Post anesthetic care unit
PEEP: Positive end expiratory pressure
PLMA: Proseal laryngeal mask airway
SADs: Supraglottic airway devices
SLMA: Supreme laryngeal mask airway
