# Erratum to: A randomized prospective controlled trial comparing the laryngeal tube suction disposable and the supreme laryngeal mask airway: the influence of head and neck position on oropharyngeal seal pressure

**DOI:** 10.1186/s12871-016-0290-2

**Published:** 2016-12-13

**Authors:** Mostafa Somri, Sonia Vaida, Gustavo Garcia Fornari, Gabriela Renee Mendoza, Pedro Charco-Mora, Naser Hawash, Ibrahim Matter, Forat Swaid, Luis Gaitini

**Affiliations:** 1 Anesthesiology Department, Bnai Zion Medical Center and Bruce and Ruth Rappaport Faculty of Medicine, Technion, Israel Institute of Technology, Haifa, Israel; 2 Surgery Department, Bnai Zion Medical Center and Bruce and Ruth Rappaport Faculty of Medicine, Technion, Israel Institute of Technology, Haifa, Israel; 3 Anesthesiology Department, Penn State College of Medicine, Hershey, PA USA; 4 Anesthesiology Department, Hospital Universitario Italiano, Buenos Aires, Argentina; 5 Anesthesiology Department, Hospital Universitario de Valencia, Valencia, Spain; 6 International Program of Teaching and Investigation in Airway Management – FIDIVA, Haifa, Israel

## Erratum

In the version of this article that was published on PubMed Central [1] the author’s name “Gustavo Garcia Fornari” was formatted incorrectly in the XML mark up and appeared incorrectly on PubMed Central. In the Mark up, “Gustavo Garcia” was added as the Given Name and “Fornari” was the Family Name, but the Family Name should have been “Garcia Fornari” and the Given Name “Gustavo”. Due to this error, the author’s name was incorrectly formatted in PubMed Central as “Fornari GG” and not as “Garcia Fornari G”. The authors name is correct on the BioMed Central website.
